# Integrated metabolomics and DNA methylation signatures and their association with flare remission in SLE

**DOI:** 10.1136/lupus-2026-002017

**Published:** 2026-09-12

**Authors:** Mary Horton, Dylan Johnson, Kirsten E Overdahl, Joanne Nititham, Kimberly E Taylor, Patricia Katz, Jimmie Ye Chun, Jinoos Yazdany, Maria Dall’Era, Lisa F Barcellos, Jian-Liang Li, Alan K Jarmusch, Lindsey A Criswell, Cristina M Lanata

**Affiliations:** 1 National Human Genome Research Institute, National Institutes of Health, Bethesda, Maryland, USA; 2 Integrative Bioinformatics Support Group, National Institute of Environmental Health Sciences, Durham, North Carolina, USA; 3 Mass Spectrometry Research Center, National Institute of Environmental Health Sciences, Durham, North Carolina, USA; 4 Division of Rheumatology, University of California San Francisco, San Francisco, California, USA; 5 Division of Epidemiology/Biostatistics, School of Public Health, University of California, Berkeley, California, USA

**Keywords:** Lupus Erythematosus, Systemic, Autoimmune Diseases, Autoimmunity

## Abstract

**Objective:**

Identify whether changes in metabolite abundance are associated with SLE flare remission and changes in DNA methylation (DNAm).

**Methods:**

40 multiethnic patients with SLE were recruited during a rheumatologist-confirmed flare and returned to the clinic 3 months later. Per visit, we obtained blood samples and generated untargeted metabolomics (ultra-high performance liquid chromatography coupled to high-resolution mass spectrometry) and DNAm (Illumina EPIC array) profiles. We identified chemical features whose change between visits was associated with remission at the follow-up visit using the *limma* package and a time × remission interaction term, adjusting for follow-up time and medications. We tested for correlations between changes in significant chemical features and DNAm at 291 sites previously associated with remission.

**Results:**

16 patients with SLE were in remission at the follow-up visit. Remitters and non-remitters had similar demographics and disease activity at baseline. We identified 62 chemical features whose changes over time were associated with remission. 19 were assigned a putative consensus chemical formula. Two were confirmed with reference standards—adenosine (interaction coefficient (β): 2.29, 95% CI 1.44 to 3.14) and bilirubin (β: 2.02, 95% CI 1.09 to 2.94). We identified 23 significantly correlated chemical feature-DNAm pairs (p<0.05/62×291). This included a strong correlation between adenosine and four DNAm sites, including three within immune-related genes*, EBF1* (r=−0.77, p=1.35×10^−7^), *IL12B* (r=0.69, p=1.87×10^−6^) and *GRAMD1C* (r=−0.71, p=1.00×10^−6^).

**Conclusion:**

Changes in chemical features, including confirmed metabolites bilirubin and adenosine, were associated with flare remission and changes in DNAm at SLE-relevant loci. Metabolites and their correlations with DNAm might serve as indicators of immune function and improve our knowledge of determinants of response to treatment. Future studies are needed to confirm the identities of metabolites and robustness of findings.

**WHAT IS ALREADY KNOWN ON THIS TOPIC**

- Understanding what drives long-term SLE outcomes and who will respond favourably to a particular treatment remains elusive. Using small chemicals (metabolites) and epigenetics might help generate novel hypotheses and insights into these mechanisms.

**WHAT THIS STUDY ADDS**

- We identified numerous chemical features that changed over time and were associated with patient remission. These included confirmed metabolites adenosine and bilirubin. Changes in adenosine and bilirubin were also correlated with DNA methylation changes in immune-relevant genes and pathways.

**HOW THIS STUDY MIGHT AFFECT RESEARCH, PRACTICE OR POLICY**

- Adenosine and bilirubin, and their correlations with DNA methylation, might serve as indicators of immune cell function and be useful for understanding long-term SLE outcomes. Further research should be done to investigate these hypotheses.

## Introduction

 SLE involves a dynamic interplay between molecular processes, particularly interferon pathways. There is a need to better understand these processes as they relate to long-term SLE outcomes given the large variability in patients’ responses to treatments and little understanding as to what drives this. Metabolites might be helpful on this front. Metabolites are small molecules present in the body from both endogenous (intermediates of metabolic pathways) and exogenous (eg, diet, microbiome, drugs) sources. There is already strong scientific support for the role of cell metabolism in driving immune cell fates and states.^1^ There is also evidence for a role of metabolites in SLE pathogenesis; metabolite levels have been identified at different levels in SLE cases compared with healthy controls.^2^ However, specific metabolite findings vary without consensus across studies. For example, levels of several amino acids, including glutamine and valine, have been shown to be lower in SLE cases compared with controls, while levels of other amino acids, such as arginine and glutamate, were higher among patients with SLE.^2^ The pathways and mechanisms by which these differential metabolites operate are poorly understood, and it is unknown whether metabolite levels are correlated with longer-term SLE outcomes or biomarkers of SLE disease activity, such as DNA methylation (DNAm).

DNAm involves the addition of a methyl group to cytosine residues in dinucleotides. This addition is intrinsically modifiable and can result from environmental and genetic factors. Patterns of DNAm have emerged as potential mechanisms for and biomarkers of SLE risk, disease activity and heterogeneity.^3–5^ Recently, we identified changes in DNAm at 291 CpG sites associated with SLE flare remission.^6^ The relationship between DNAm and metabolites is complex and bidirectional and is still being understood.^7^ Ultimately, metabolites and their correlations with DNAm might serve as indicators of immune cell function and improve our knowledge of determinants of response to treatment in the future.

In this exploratory study, we used 40 multiethnic patients with SLE recruited during an active flare who had untargeted metabolomics and DNAm profiles generated at baseline flare and approximately 3 months later. Our objectives were to: (1) identify whether changes in metabolite abundances between baseline flare and follow-up were associated with complete flare remission at follow-up, and (2) identify whether metabolite changes associated with remission were correlated with changes in DNAm at 291 sites previously associated with flare remission.

## Patients and methods

### Study subjects and design

Individuals experiencing an active SLE flare were recruited to the California Lupus Epidemiology Study Flare Cohort during routine clinical care at the University of California, San Francisco (UCSF). SLE diagnoses were confirmed by study physicians based on one of the following definitions: (1) meeting ≥4 of the 11 American College of Rheumatology (ACR) revised criteria for the classification of SLE as defined in 1982 and updated in 1997, (2) meeting 3 of the 11 ACR criteria plus a documented rheumatologist’s diagnosis of SLE or (3) a confirmed diagnosis of lupus nephritis, defined as fulfilling the ACR renal classification criterion (>0.5 g of proteinuria per day or 3+ protein on urine dipstick analysis) or having evidence of lupus nephritis on kidney biopsy. A flare was established by the treating physician and characterised by the Safety of Estrogens in Lupus Erythematosus: National Assessment trial (SELENA) SLE Disease Activity Index (SLEDAI).^8^ Requirements for inclusion included a SLEDAI score ≥3 and a physician’s determination that a flare was significant enough to warrant a treatment change. Approximately 3 months later, participants returned to the clinic as part of routine clinical care. Individuals with blood samples and complete clinical data at each visit were included in our sample. This resulted in a total of 122 paired samples (61 individuals).

Neither patients nor the public were involved in the design, conduct, reporting or dissemination plans of this research.

### Untargeted metabolomics data

A random subset of 40 subjects (80 paired samples) who had DNAm data already generated and were included in a previous study were selected for untargeted metabolomics performed by the National Institutes of Health Metabolomics Core.^6^ Plasma samples from each visit were prepared for analysis with a 4:1 v/v methanol crash. Samples were analysed using ultra-high performance liquid chromatography (LC) coupled to high-resolution mass spectrometry (MS). LC-MS data were acquired for each sample. LC-MS/MS data were acquired for annotation via AcquireX on replicate injections of the pooled quality control (QC). Ionisation was performed via heated electrospray ionisation. Data were collected in both positive and negative ionisation modes. To minimise batch effects, samples were analysed in a single acquisition batch in random sample order (randomised according to study participants, study visit and sample preparation time). No batch or run order effects were noted by principal component analysis or visual examination of the data. The pooled QC was injected in triplicate at multiple volumes to assess signal response proportionality as part of quality assurance. Features were defined as a pairing of a mass-to-charge ratio (*m/z*) with a corresponding retention time (RT). Features were annotated via spectral database matches to public and commercial libraries (GNPS, NIST, mzCloud, MassBank) as well as in-house MS/MS spectral libraries. A detailed protocol of sample processing and analysis is included in online supplemental materials. Compound Discoverer 3.3.0.550 (Thermo Scientific) was used to process .raw files in a tabular output, including descriptors of each feature, annotation information (eg, MS/MS database match) and peak area. Features were annotated via spectral database matches to public, commercial and in-house MS/MS spectral libraries.

A total of 10 150 features in positive mode and 11 553 features in negative mode were identified. Features that were undetected or below the limit of detection in > 50% of all samples were excluded from the final analysis. Raw feature abundance was log2-transformed and median-centred within each sample by applying a log-ratio shift, subtracting each sample’s median log2 count. This procedure aligned sample medians to zero while preserving within-sample rank structure. Features with poor proportionality of response (Spearman correlation p>0.05) or large dispersion ratio (D-ratio >50%) were filtered out of the dataset.^9 10^ Following filtering, 4055 features in positive mode and 4921 features in negative mode were used in analyses. Features were initially annotated via *m/z* and RT matching to an in-house database of authentic reference standards, as well as via *m/z* and MS/MS matching to public and commercially-available spectral databases (including NIST2020, GNPS, mzCloud and in-house MS/MS spectral library acquired from authentic chemical standards purchased and run by the Mass Spectrometry Research Center). Features with a database or library match were putatively annotated. In accordance with the Metabolomics Standards Initiative (MSI), MSI levels were used to convey confidence levels of each annotation.^11^ Confidence levels were distinguished depending on whether an annotation was confirmed with an authentic reference standard (either MSI Level 1: *m/z*, MS/MS and RT match to reference standard or MSI Level 2: *m/z* and RT matches to reference standards) or via database matches (MSI Level 2: *m/z* and MS/MS). Chemical annotations were manually curated as appropriate (eg, checking that chemical names and masses match, checking for spelling or notation errors).

Following statistical analyses (detailed below), for any significant feature not originally successfully annotated via MS/MS database matching, we assigned a putative weight-of-evidence annotation where possible. For all features with MS/MS data, probable molecular formulae were predicted in SIRIUS via fragmentation tree scoring (specific parameters for computing predicted molecular formulae can be found in suplementary information (SI)). ^12 13^ Molecular formulae were also assigned based on MS1 (parent ion) matches in the Human Metabolome Database (HMDB)^14 15^ via the LC-MS spectra search (specific parameters can be found in SI). Per feature, possible HMDB-identified metabolites were queried for their chemical taxonomy using ClassyFire^16^, and chemical class and subclass were assigned to each candidate metabolite. For features where SIRIUS and HMDB predictions produced the same chemical formula prediction, a consensus molecular formula was assigned to the feature. Where possible, a consensus ClassyFire taxonomy was assigned to the molecular formula based on the most-represented chemical class and subclass among the HMDB-identified metabolites for that formula; for features where no candidate HMDB-identified metabolites had shared chemical taxonomy, no chemical class and/or subclass were assigned (represented as ‘no consensus’). Where only a SIRIUS formula prediction or only an HMDB prediction was available, or where SIRIUS and HMDB predictions were both available but produced no consensus, no formula was assigned. For features where the putative consensus formula matched the formula and RT of an authentic reference standard, a metabolite name was assigned to the feature (MSI Level 1).

### DNA methylation data

Details were previously described.^6^ Briefly, genome-wide DNAm was measured using the Illumina Infinium MethylationEPIC BeadChip V.1.0 from DNA extracted from whole blood at each visit. DNAm data were processed using the *minfi* package in R V.4.3.3.^17 18^ Signal intensities were background-subtracted using the noob function and quantile-normalised.

Global cell-type proportions for 12 leucocyte subtypes (neutrophils, eosinophils, basophils, monocytes, naive and memory B cells, naive and memory CD4+ and CD8 + T cells, natural killer and T regulatory cells) were estimated at each visit using *estimateCellCounts2*() with EPIC IDOL-Ext library CpGs in the *FlowSorted.Blood.EPIC* package in *R*.^19^

### Clinical data

Clinic visits included the collection and review of medical records, a SLE history and physical examination conducted by a physician specialising in SLE, and completion of a structured interview by an experienced research assistant. Data collected included sex, self-reported race, Hispanic ethnicity, age and SELENA SLEDAI components and score at each visit. Medication assessment included whether individuals were on the following at the time of each blood draw: prednisone, cyclophosphamide, mycophenolate mofetil (MMF) or mycophenolic acid (MPA), rituximab, belimumab, azathioprine, cyclosporine, tacrolimus, hydroxychloroquine, methotrexate and solumedrol. One person had missing medication for the follow-up visit, so their medications were imputed with the baseline visit medications. Multiple correspondence analysis (MCA), similar to principal component analysis but for categorical variables, was conducted separately at each time point on binary medication variables to reduce the dimensionality of the medication data using the *FactoMineR* R package.^20^

### Statistical analyses

Our primary objective was to identify chemical (metabolomic) features where a change in abundance was associated with complete remission at follow-up. We conservatively defined ‘remitter’ as a patient having a SELENA SLEDAI score of 0 at the follow-up visit. For each chemical feature that reached QC thresholds, we used a linear model with a *time* × *remission* interaction term in the *limma* R package.^21^ Time represented baseline flare (time=0) or follow-up (time=1) visits. The paired design was accounted for using the *duplicatecorrelation*() function. Interaction coefficients were interpreted as the average difference in change in chemical feature abundance between baseline and follow-up, comparing the remitters to non-remitters. Model covariates included days between visits and the first medication MCA component. Covariates were selected based on a data-driven approach, given the small number of samples in any individual covariate category. Characteristics such as sex, ethnicity and age at diagnosis were not included because the paired longitudinal design reduces concerns about confounding by unchanging subject-level characteristics. As a sanity check, we confirmed these variables were not associated with remission status using bivariate logistic or linear regression models (p<0.05). Additional medication MCA components were also not included because they were not associated with remission status. Analyses were conducted separately for positive and negative mode features. Per mode, features with a false discovery rate (FDR) *q*<0.05 were considered significant.

Our group previously identified that changes in DNAm at 1953 CpG sites were associated with SLE remission using similar approaches to the current analysis, including study design, subjects (59 paired samples instead of 40) and statistical models.^6^ These were largely located in interferon-response genes and immune-related pathways. For the present study, we used 291 of these CpGs (those with large effect sizes, at least a 10% difference in change in DNAm between study visits between remitters and non-remitters) to test whether changes in DNAm and changes in significant remission-associated metabolites were correlated. Changes were defined as the standardised difference in DNAm beta-values (or chemical features abundance) between baseline flare and follow-up visits. DNAm-chemical feature pairs were tested using Spearman correlations, and those with Bonferroni p<0.05/(#significant metabolites × 291 CpGs) were considered significant.

## Results

### Characteristics of participants

16 participants fully remitted by the follow-up visit (table 1). Remitters and non-remitters did not have statistically significantly different sex, race, ethnicity, age or age at diagnosis. SLEDAI at the baseline flare was similar among remitters and non-remitters (mean=11.7 (SD=5.3) and 12.2 (SD=6.3), respectively). At the follow-up visit, non-remitters had a mean SLEDAI=6.0 (SD=4.9). At baseline, remitters and non-remitters did not have significantly different proportions of SLEDAI proteinuria (44% and 50%, respectively), an indicator of lupus nephritis. All SLEDAI components at baseline and estimated cell type proportions were similar among remitters and non-remitters, except for CD8+ naïve T-cells at baseline and follow-up and natural killer cells at baseline, which were higher among non-remitters (online supplemental table 1). Patients were not treatment-naïve at baseline, and medication use at follow-up was similar between remitters and non-remitters (table 1).

**Table 1:** Clinical and demographic features of 40 SLE participants by remission status at follow-up visit

	Remission status		
	Yes	No	P value
N	16	24	
Female, n (%)	15 (93.8)	21 (87.5)	0.91
Self-reported race, n (%)			0.75
Asian	5 (55.6)	7 (41.2)	
Black	2 (22.2)	3 (17.6)	
White	1 (11.1)	5 (29.4)	
Other	0 (0.00)	1 (5.9)	
Multiple races^*^	1 (11.1)	1 (5.9)	
Not reported†	7 (43.8)	6 (25.0)	
Hispanic, n (%)	7 (43.8)	7 (29.2)	0.54
Age at diagnosis (years), mean (SD)	32.1 (15.1)	27.0 (10.4)	0.21
Age at baseline flare visit (years), mean (SD)	38.1 (13.1)	35.3 (10.5)	0.46
Days between visits, median (IQR)	127.0 (58.8–290.5)	77.0 (43.3–127.0)	0.10
SELENA SLEDAI, mean (SD)			
Baseline	11.7 (5.3)	12.2 (6.3)	0.80
Follow-up	0.00 (0.00)	6.0 (4.9)	<0.001
SLEDAI proteinuria, n (%)			
Baseline	7 (43.8)	12 (50.0)	0.95
Follow-up	0 (0.00)	4 (16.7)	0.24
Medications at follow-up (yes/no), n (%)			
Prednisone	12 (75.0)	18 (75.0)	1.00
Hydroxychloroquine	15 (93.8)	21 (87.5)	0.91
Mycophenolate	6 (37.5)	8 (33.3)	1.00
Azathioprine	0 (0.00)	6 (25.0)	0.09
Belimumab	1 (6.2)	3 (12.5)	0.91
Cyclophosphamide	1 (6.2)	2 (8.3)	1.00
Solumedrol	1 (6.2)	0 (0.00)	0.84
Rituximab	1 (6.2)	2 (8.3)	1.00
Tacrolimus	0 (0.00)	2 (8.3)	0.66
Methotrexate	2 (12.5)	2 (8.3)	1.00

Remission is defined as SLEDAI=0 at follow-up visit.

* Additional race categories not solely indicated by participants included American Indian and Pacific Islander.

† Majority of participants not reporting race identified as Hispanic.

SELENA SLEDAI, SLE Disease Activity Index-the Safety of Estrogens in Lupus Erythematosus: National Assessment trial.

### Changes in metabolite abundances were associated with SLE remission status

**Figure 1:**
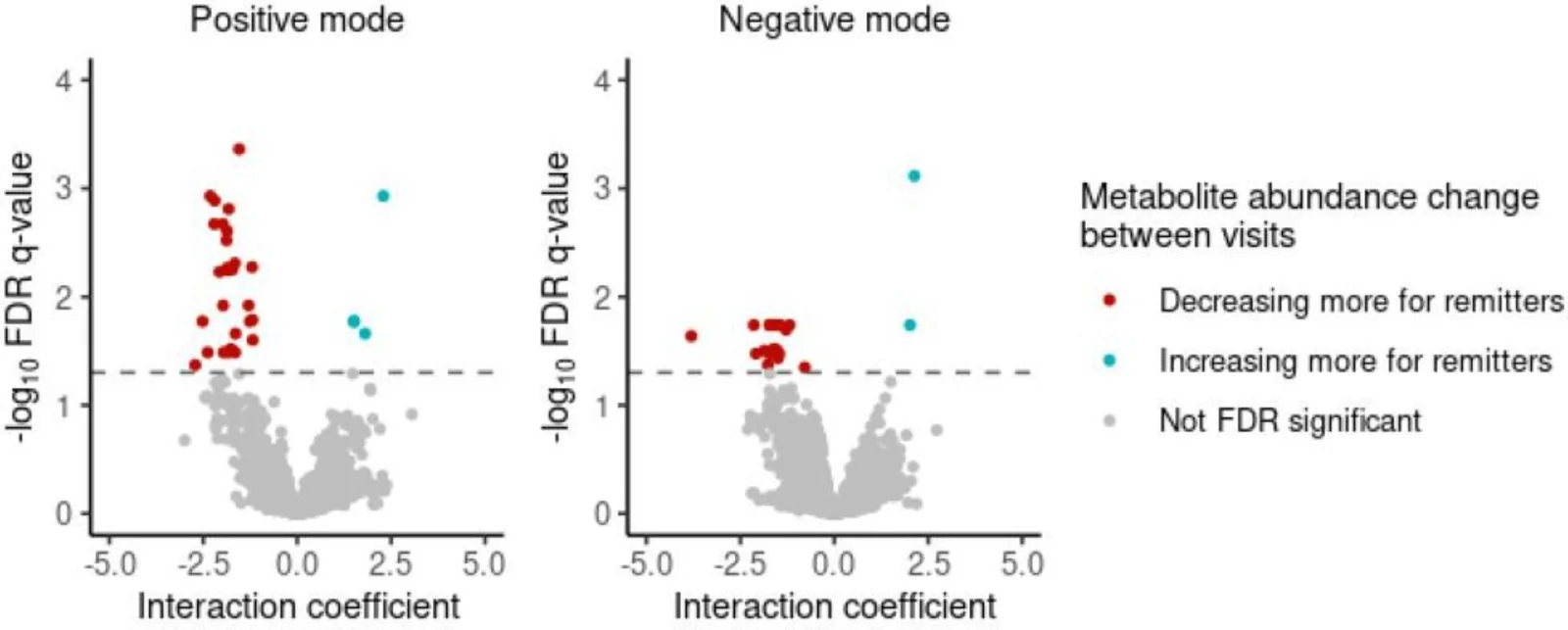
Changes in chemical (metabolite) feature abundances among active patients with SLE were associated with remission status. Positive and negative mode metabolites are shown separately. Coloured metabolites had changes over time that were significantly associated with remission at FDR *q*<0.05. Dotted line indicates FDR *q*=0.05. FDR, false discovery rate.

**Figure 2:**
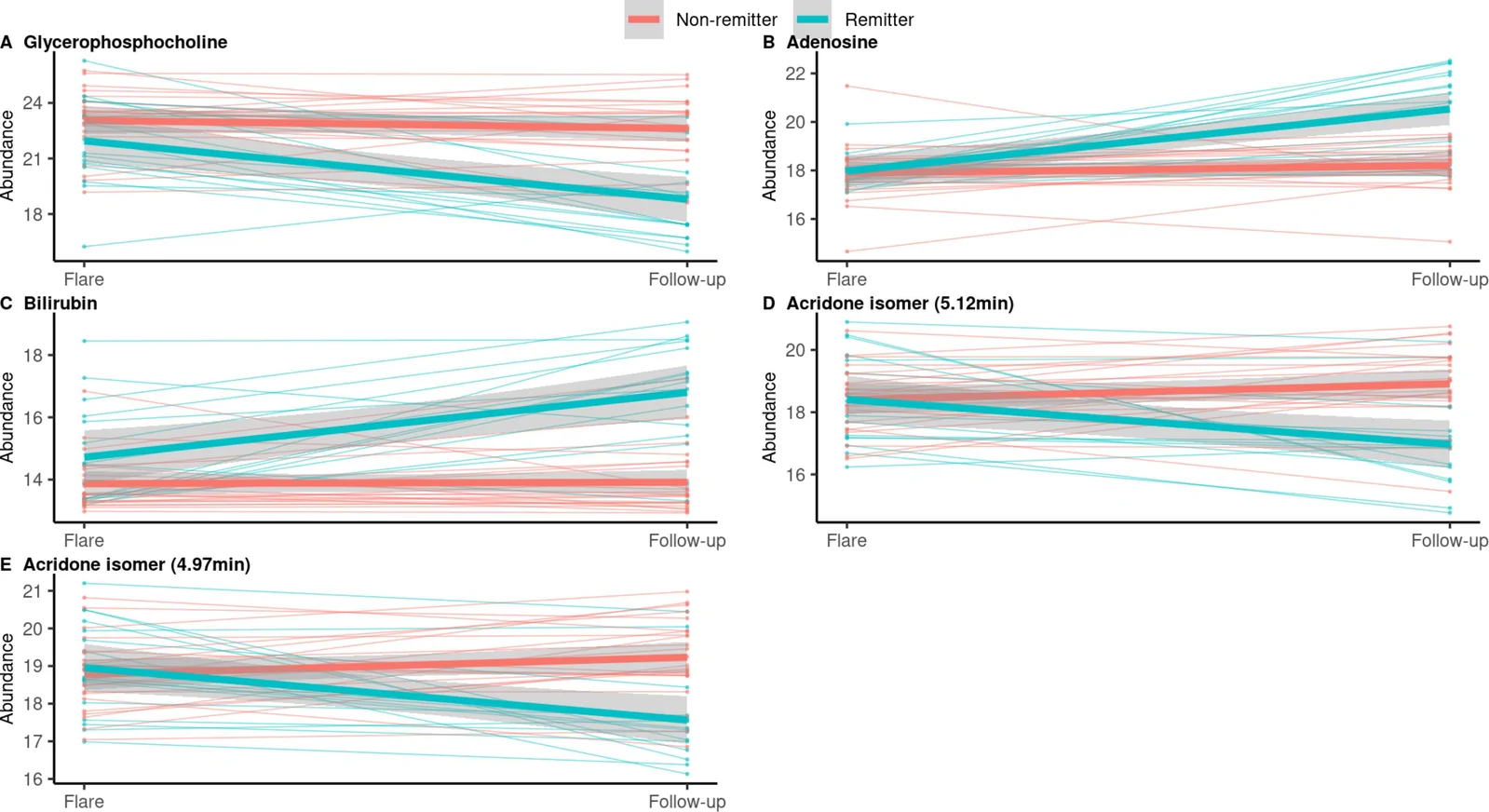
Distributions of five significant and putatively annotated metabolites with the largest effect sizes. Metabolite abundance trajectories for remitters and non-remitters for (**A**) glycerophosphocholine, (**B**) adenosine, (**C**) bilirubin and (**D and E**) two isomers of acridone. Bolded lines indicate the line of best fit stratified by remission status. Grey bars indicate SEs.

Our primary objective was to identify chemical features (named by unique *m/z* and RT pairing) with changes in abundance between baseline flare and follow-up visits that were associated with remission status. We identified 62 significant remission-associated features (FDR *q*<0.05) (figure 1, online supplemental table 2). For 92% of these features (n=56), abundances decreased from flare to follow-up visit among remitters (mean change of −1.02) but were unchanged or slightly increased between visits for non-remitters (mean change of 0.48). After annotating significant features, consensus formulae were assigned to 19 features (table 2). The five significant metabolites with the largest effect sizes included a glycerophosphocholine (MSI Level 3, 774.5643@9.32 min., interaction coefficient (β): −2.71, 95% CI −4.15 to −1.27), adenosine (MSI Level 1, 268.1040@3.34 min., β: 2.29, 95% CI 1.44 to 3.14), bilirubin (MSI Level 1, 583.2564@7.16 min., β 2.02, 95% CI: 1.09 to 2.94) and two acridone isomers (MSI Level 3, 302.1023@5.12 and 4.97 min., respectively, β: −1.97, 95% CI −2.89 to −1.06 and β: −1.88, 95% CI −2.66 to −1.11). For these five metabolites, patients’ respective abundance trajectories between visits are shown in figure 2.

**Table 2:** Chemical features (*m/z* and RT) whose changes were significantly associated with flare remission (FDR <0.05) and were assigned putative weight-of-evidence annotations

Feature (*m/z* and RT)	MSI confidence level*	Confirmed annotations†	Consensus molecular formula‡	Putative chemical class§	Putative chemical subclass§	Interaction coefficient	95% CI	FDR *q*
Positive Ionisation mode								
Features with consensus molecular formula, putative chemical class, putative chemical subclass and confirmed with authentic reference standard								
268.1040 @ 3.34 min.	MSI Level 1	Adenosine	C_10_H_13_N_5_O_4_	Purine nucleosides	Purine ribonucleosides	2.29	1.44 to 3.14	1.17e-03
Features with consensus molecular formula, putative chemical class and putative chemical subclass								
247.1288¶ @ 1.88 min.	MSI Level 3		C_10_H_18_N_2_O_5_	Carboxylic acids and derivatives	Amino acids, peptides and analogues	−1.77	−2.67 to −0.86	3.06e-02
302.1023 @ 4.97 min.	MSI Level 3		C_16_H_15_NO_5_	Quinolines and derivatives	Benzoquinolines: Acridones	−1.88	−2.66 to −1.11	3.01e-03
302.1023 @ 5.12 min.	MSI Level 3		C_16_H_15_NO_5_	Quinolines and derivatives	Benzoquinolines: Acridones	−1.97	−2.89 to −1.06	1.20e-02
302.1022 @ 4.8 min.	MSI Level 3		C_16_H_15_NO_5_	Quinolines and derivatives	Benzoquinolines: Acridones	−1.64	−2.46 to −0.83	2.19e-02
774.5643 @ 9.32 min.	MSI Level 3		C_42_H_80_NO_9_P	Glycerophospholipids	Glycerophosphocholines	−2.71	−4.15 to −1.27	4.25e-02
Features with consensus molecular formula and putative chemical class								
315.134 @ 5.92 min.	MSI Level 3		C_17_H_18_N_2_O_4_	Azoles/Indoles	(No consensus)	−1.29	−1.89 to −0.70	1.20e-02
315.134 @ 6.08 min.	MSI Level 3		C_17_H_18_N_2_O_4_	Azoles/Indoles	(No consensus)	−1.19	−1.76 to −0.63	1.63e-02
283.1077 @ 4.95 min.	MSI Level 3		C_16_H_14_N_2_O_3_	Benzopyrazoles or Azolidines	(No consensus)	−1.82	−2.76 to −0.88	3.26e-02
Features with consensus molecular formula								
301.1183 @ 4.96 min.	MSI Level 3		C_16_H_16_N_2_O_4_	(No consensus)	(No consensus)	−1.87	−2.63 to −1.11	2.47e-03
301.1183 @ 5.12 min.	MSI Level 3		C_16_H_16_N_2_O_4_	(No consensus)	(No consensus)	−1.86	−2.67 to −1.06	5.42e-03
Negative Ionisation Mode								
Features with consensus molecular formula, putative chemical class, putative chemical subclass and confirmation with authentic reference standard								
583.2564 @ 7.16 min.	MSI Level 1	Bilirubin	C_33_H_36_N_4_O_6_	Tetrapyrroles and derivatives	Bilirubins	2.02	1.09 to 2.94	1.82e-02
Features with consensus molecular formula, putative chemical class and putative chemical subclass								
273.1243 @ 5.12 min.	MSI Level 3		C_15_H_18_N_2_O_3_	Carboxylic acids and derivatives	Amino acids, peptides and analogues	−1.65	−2.45 to −0.84	3.12e-02
273.1243 @ 4.81 min.	MSI Level 3		C_15_H_18_N_2_O_3_	Carboxylic acids and derivatives	Amino acids, peptides and analogues	−1.6	−2.32 to −0.88	1.82e-02
273.1244 @ 4.98 min.	MSI Level 3		C_15_H_18_N_2_O_3_	Carboxylic acids and derivatives	Amino acids, peptides and analogues	−1.57	−2.29 to −0.85	1.82e-02
349.096 @ 6.5 min.	MSI Level 3		C_15_H_18_N_4_O_4_S	Lactams	Beta lactams	−1.64	−2.46 to −0.82	3.14e-02
349.096 @ 6.3 min.	MSI Level 3		C_15_H_18_N_4_O_4_S	Lactams	Beta lactams	−1.47	−2.21 to −0.73	3.34e-02
Features with consensus molecular formula and putative chemical class								
255.1139 @ 5.13 min.	MSI Level 3		C_15_H_16_N_2_O_2_	Benzenes/Anisoles	(No consensus)	−1.59	−2.38 to −0.80	3.12e-02
255.1139 @ 4.98 min.	MSI Level 3		C_15_H_16_N_2_O_2_	Benzenes/Anisoles	(No consensus)	−1.53	−2.20 to −0.85	1.82e-02

* Confidence levels were assigned based on the MSI criteria.

† Where possible, putative weight of evidence annotations were confirmed with an authentic reference standard.

‡ Consensus molecular formula assigned via HMDB *m/z* matching and SIRIUS MS/MS prediction.

§ Most represented ClassyFire results based on HMDB *m/z* matching.

¶ One of two features was retained because both had identical *m/z *and nearly identical retention times (188 and 187), so they were likely to be the same metabolite.

FDR, false discovery rate; HMDB, Human Metabolome Database; MS, mass spectrometry; MSI, Metabolomics Standards Initiative; m/z, mass-to-charge ratio; RT, retention time.

### Changes in metabolites are correlated with changes in DNA methylation

**Figure 3:**
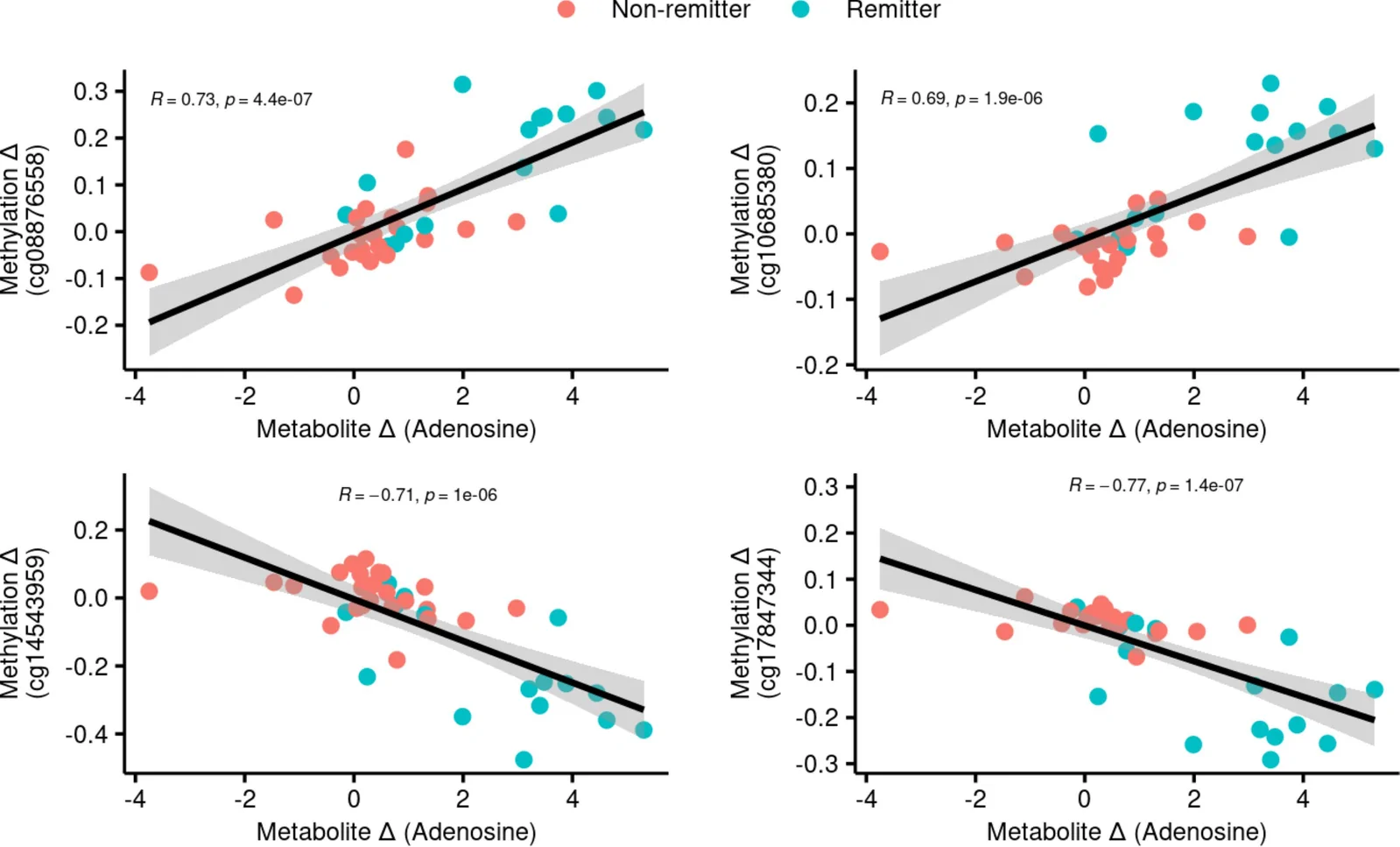
Correlations between change in adenosine abundance and change in DNA methylation at four CpG sites for patients with SLE with active disease who did (blue) and did not (red) remit from flare to postflare visits. Negative change represents a lower value at follow-up compared with the flare visit, and positive change represents a higher value at follow-up. Spearman correlation coefficients and p values are indicated.

We tested whether changes in the 62 significant remission-associated chemical features were correlated with changes in DNAm at 291 CpG sites previously identified as associated with remission. We identified 23 significantly correlated chemical feature-DNAm change pairs (p<0.05/ (62x291)). This included 11 chemical features and 16 CpG sites (table 3). Many CpG sites were within the promoter or body of genes related to immune function, including *IL12B, EBF1* and *AIM2*. These genes encode a cytokine that acts on T and natural killer cells, encodes a B cell transcription factor and recognises double-stranded DNA, which triggers the inflammasome, respectively. There were several instances where a change in DNAm at a single CpG site was correlated with changes in multiple chemical features. For example, a change in cg10685380, within the body of *IL12B*, was correlated with changes in three chemical features. One of these was confirmed as adenosine using a reference standard (268.1040@3.34 min., correlation=0.69, p=1.87×10^−6^). Three other chemical features, including an azole/indole (315.134@5.92 min., MSI Level 3, correlation=−0.70, p=1.18×10^−6^), were associated with DNAm change in a single CpG site, cg15004555, within the body of *AIM2*. In contrast, there were several instances where a change in abundance of a single chemical feature was correlated with changes in DNAm at multiple CpG sites. For example, as shown in figure 3, changes in adenosine (268.1040@3.34 min.) were strongly correlated with changes in DNAm at four CpG sites, including CpGs within the body of *EBF1* (correlation=−0.77, p=1.35×10^−7^) and within 200 bp of the transcription start site of *GRAMD1C* (encodes a cholesterol transporter, correlation=−0.71, p=1.00×10^−6^).

**Table 3:** Bonferroni significant (p<0.05/(62x291)) correlations between changes in chemical features and DNA methylation

Feature (*m/z* and RT)	CpG	P value	Correlation	Chr:position	Gene	Location	Gene’s relevance to immune function, SLE or autoimmunity
MSI level 1–3 features (as shown in table 2: confirmed or putatively annotated)							
247.1288 @ 1.88* min.	cg26271086	3.19e-07	0.74	14:39901518	*FBXO33*	1stExon; 5'UTR	
268.1040 @ 3.34 min.	cg08876558	4.43e-07	0.73	11:59394462			
268.1040 @ 3.34 min.	cg10685380	1.87e-06	0.69	5:158745090	*IL12B*	Body	Encodes a subunit of IL-12, a cytokine that acts on T and natural killer cells. IL-12 is higher in SLE cases compared with controls and is associated with SLE disease activity. PMID: 10209522
268.1040 @ 3.34 min.	cg14543959	1.00e-06	−0.71	3:113557658	*GRAMD1C*	TSS200	Expression associated with IL-6 levels. PMID: 25311648
268.1040 @ 3.34 min.	cg17847344	1.35e-07	−0.77	5:158478734	*EBF1*	Body	B-cell transcription factor. EBF1 binding sites are enriched in SLE-associated CpGs near IFN-regulated genes. PMID: 31430064
301.0958 @ 4.80 min.	cg04667820	2.33e-06	−0.69	2:64218766	*VPS54*	5'UTR	B-cell expression associated with SLE
315.134 @ 5.92 min.	cg15004555	1.18e-06	−0.70	1:159043116	*AIM2*	Body	Recognises dsDNA and triggers the formation of the AIM2 inflammasome
MSI level 4 features (not able to be annotated; see online supplemental table 2)							
134.0474 @ 3.33 min.	cg04792380	7.07e-07	0.72	12:105162318			
134.0474 @ 3.33 min.	cg10495947	2.63e-06	−0.68	3:39148675	*TTC21A; GORASP1*	TSS1500; Body	Associated with immune-related signalling. PMID:38151293
134.0474 @ 3.33 min.	cg10685380	1.52e-06	0.70	5:158745090	*IL12B*	Body	Encodes a subunit of IL-12, a cytokine that acts on T and natural killer cells. IL-12 is higher in SLE cases compared with controls and is associated with SLE disease activity. PMID: 10209522
134.0474 @ 3.33 min.	cg16441688	1.99e-06	0.69	1:38042075	*GNL2*	Body	
134.0474 @ 3.33 min.	cg17847344	2.88e-07	−0.74	5:158478734	*EBF1*	Body	B-cell transcription factor. EBF1 binding sites are enriched in SLE-associated CpGs near IFN-regulated genes. PMID: 31430064
136.0617 @ 3.34 min.	cg10685380	1.82e-06	0.69	5:158745090	*IL12B*	Body	Encodes a subunit of IL-12, a cytokine that acts on T and natural killer cells. IL-12 is higher in SLE cases compared with controls and is associated with SLE disease activity. PMID: 10209522
219.1338 @ 0.58 min.	cg02187666	2.65e-06	−0.68	6:161238481			
219.1338 @ 0.58 min.	cg15004555	1.90e-06	−0.69	1:159043116	*AIM2*	Body	Recognises dsDNA and triggers the formation of the AIM2 inflammasome
277.0862 @ 2.01 min.	cg04868116	1.09e-06	−0.70	10:53401405	*PRKG1*	Body	Associated with serum IFN-α activity in SLE cases. PMCID: PMC4305028
277.0862 @ 2.01 min.	cg09615453	1.71e-06	0.69	17:48351743	*TMEM92*	TSS200; 5'UTR	
277.0862 @ 2.01 min.	cg12045156	8.12e-07	−0.71	17:67519707	*MAP2K6*	Body	Regulates inflammation and apoptosis. PMID: 40378928
299.0682 @ 1.60 min.	cg04868116	1.07e-06	−0.70	10:53401405	*PRKG1*	Body	Associated with serum IFN-α activity in SLE cases. PMCID: PMC4305028
299.0682 @ 1.60 min.	cg09615453	2.40e-06	0.68	17:48351743	*TMEM92*	TSS200; 5'UTR	
313.1194 @ 5.92 min.	cg11983363	4.21e-07	−0.73	2:32578646			
313.1194 @ 5.92 min.	cg15004555	1.21e-06	−0.70	1:159043116	*AIM2*	Body	Recognises dsDNA and triggers the formation of the AIM2 inflammasome
440.1463 @ 5.83 min.	cg17502989	2.67e-06	0.68	6:153451486	*RGS17*	5'UTR	Interacts with proinflammatory mediators, including CXCL1, a strong marker of SLE disease activity. PMID: 40061942

* One of two features was retained because both had identical *m/z *and nearly identical retention times (188 and 187), so they were likely to be the same metabolite.

chr, chromosome; dsDNA, double-stranded DNA; IFN, interferon; IL, interleukin; MSI, Metabolomics Standards Initiative; m/z, mass-to-charge ratio; RT, retention time; TSS, transcription start site; UTR, untranslated region.

## Discussion

Using untargeted metabolomics from a cohort of patients with SLE with active disease, we identified 62 chemical features whose change over time significantly differed depending on whether a patient fully remitted at follow-up or not. Two of these, adenosine and bilirubin, were confirmed with authentic reference standards (MSI Level 1); 19 features were putatively annotated with a chemical formula. More than half of the putatively annotated features shared an m/z with at least one other feature but had different retention times, potentially indicating isomers. Generally, chemical feature abundance decreased from flare to follow-up for remitters but remained unchanged for non-remitters. However, for bilirubin and adenosine, abundance increased over time for remitters. Both have previously been linked to anti-inflammatory processes and SLE. We also identified 23 CpG-chemical feature pairs with significantly correlated changes between flare and follow-up visits. This included correlations between change in adenosine and changes in DNAm at four CpG sites located in genes relevant to immune function. Overall, these findings highlight several chemical features and mechanisms to further investigate which may help us better understand the underlying biology of active SLE and response to treatment. Below, we highlight several interesting findings given their previous associations with SLE or potential role in autoimmunity. Future studies are needed to independently validate these findings.

Our study showed that bilirubin levels increased for remitters between flare and follow-up but remained unchanged for non-remitters. Bilirubin is the natural end-product of heme metabolism and a powerful antioxidant. A meta-analysis of five studies by Yu *et al*^22^ found that serum bilirubin was nearly 40% lower among SLE cases compared with healthy controls.^22^ This meta-analysis also showed a consistent relationship between higher bilirubin and lower disease severity scores and less multiorgan involvement. Bilirubin has many functions potentially relevant for our observed association, including influencing the activity of regulatory T (Treg) and T helper 17 (Th17) cells, deactivating the NLRP3 inflammasome, hindering the Toll‐like receptor 4/nuclear factor kappa B signalling pathway and inhibiting the expression of complement activation fragment 5 a receptor one.^23–28^ While current research on bilirubin and SLE is limited, the agreement of our findings suggests that more work should be done to understand its role in reducing disease activity.

Our study also found that adenosine, which is crucial for immune homeostasis and preventing immune system hyperactivity, increased for remitters between flare and follow-up but remained unchanged for non-remitters. Few studies have investigated the association between adenosine and SLE. Of those that have, adenosine was lower in the serum and stool of SLE cases compared with controls.^29–31^ This generally protective effect of adenosine on SLE agrees with our results that remitters had higher adenosine levels at the postflare visit compared with non-remitters. Adenosine is known to modulate inflammatory responses.^32 33^ Extracellular adenosine is detected by receptors present on nearly all immune cells, which leads to its widespread use in regulating the immune system, including balancing the distribution of anti-inflammatory Tregs and pro-inflammatory Th17 cells.^34^

Acridones, a chemical subclass putatively assigned to three of this study’s significant chemical features, are derivatives of MPA, the active form of MMF, which is an important immunosuppressive medication for treating lupus nephritis.^35^ Acridone derivatives, especially those conjugated with MPA, have been investigated for their ability to inhibit inosine-5’-monophosphate dehydrogenase (IMPDH).^36^ IMPDH is a key enzyme in the de novo synthesis of guanosine nucleotides, which are essential for the proliferation of T and B lymphocytes, making it a target for immunosuppressive agents. In our results, acridone-annotated metabolites decreased from flare to follow-up for remitters and were relatively unchanged for non-remitters. Overall, 38% of remitters and 33% of non-remitters were using MMF at follow-up. Given the decrease in acridone abundance among remitters, a modest overall percentage of MMF used by participants and a small difference in MMF use between remitters and non-remitters, it seems unlikely that switching to MMF was driving the observed association between acridone and remission. More research should be done to understand the potential role of acridone and investigate sources of acridone apart from MPA.

Our study found that 11 significant remission-associated chemical features were correlated with changes in DNAm at 16 CpG sites previously identified as associated with remission (23 total significant chemical-DNAm correlations). Most of these CpG sites were located within the promoter or body of genes related to immune function, including *IL12B, EBF1* and *AIM2*. This is not surprising, given these CpG sites were previously identified as associated with flare remission. Interestingly, changes in adenosine were strongly correlated with changes in DNAm at four CpG sites. This included CpGs within the body of *EBF1* (encodes a B cell transcription factor), within 200 bp of the transcription start site of *GRAMD1C* (encodes a cholesterol transporter) and within the body of *IL12B* (involved in the differentiation of naive T cells into Th1 cells and induces interferon production). Adenosine was previously found to inhibit IL12.^37^ In our study, changes in adenosine were positively correlated with changes in DNA methylation at cg10685380 within the IL12B gene body, suggesting a potential link between adenosine signaling and epigenetic regulation of an SLE-relevant immune pathway. Adenosine can be an intermediate in DNAm.^38^ During DNA methylation, S-adenosylmethionine (SAM) donates a methyl group and is converted to S-adenosylhomocysteine (SAH), which can subsequently be metabolised to adenosine and homocysteine. Adenosine is critical in many functions including energy metabolism, inflammation and DNAm. In this study, the causative nature of adenosine cannot be determined due to the variety of roles it can play. It is also possible that the observed DNAm-metabolites correlations are driven by cell type composition and linked to a shared upstream factor. This may be considered a confounder or variable of interest given that DNAm can drive cell identity. More information is needed to establish the direction of our observed association which may be confounded by other factors in the biochemical network. In addition, these results should be interpreted as exploratory and should not be viewed as independent validation of the metabolomic findings. Future work should be done to consider the potential biological link between flare remission, DNAm, cell type proportions and metabolites, particularly adenosine.

Untargeted metabolomics is an unbiased method for discovering novel metabolites or biomarkers of flare remission; however, annotating the chemical features remains challenging. Spectral database matching as a form of annotation is limited by prior knowledge and inputs into databases. Our weight-of-evidence putative annotation approach presents a method by which to overcome these challenges and gain high-confidence biological insight into a greater number of significant features. However, even when using best practices for untargeted feature annotation, over 75% of our significant remission-associated chemical features could not be annotated with a chemical formula. This lack of annotation can present frustrations where empirical mass spectrometry and DNAm measurements show clear correlations. For example, in our chemical feature-DNAm change correlation results, changes in five CpG sites were correlated with changes in one unnamed chemical feature (134.0474@3.3 min.). These five CpGs are in immune-relevant genes, including *EBF1* (a B cell transcription factor) and *IL12B*. Additionally, DNAm change at a CpG within *IL12B*, which we previously discussed in correlation with change in adenosine, was also correlated with changes in two unnamed chemical features (134.0474@3.33 min. and 136.0617@3.34 min.). Future research should be done to confirm MSI Level 3 putative annotations with authentic reference standards (ie, targeted validation via LC-MS/MS) and to resolve identities of unannotated features in order to understand their connections to DNAm and flare remission. Because untargeted metabolomics is intended to be a discovery-based tool, we hope the results presented are a first step toward refining more targeted experiments that narrow the scope of chemical space and further explore these yet-unknown metabolites.

This study has several additional strengths and limitations. Our untargeted metabolomics approach is a strength, given the limited prior knowledge about metabolites associated with SLE or drivers of flare remission. It provides hypotheses for future studies that should be investigated. However, the limitation of this approach is that many annotations are putative, and assay results do not generate absolute quantification information. Another strength is that our study has two timepoints and only includes individuals actively experiencing a physician-diagnosed flare and warranting a treatment change at study enrolment. This sheds light on mechanisms underpinning active disease. This contrasts with most SLE studies that are cross-sectional and include patients with relatively low disease activity. Despite this design, it was still not possible to determine the temporality of correlated chemical feature-DNAm pairs, that is, whether metabolites caused changes in DNA or vice versa. More blood samples taken at shorter intervals would be necessary for this. Additionally, our study involved a real-world uncontrolled medication setting, making it difficult to study treatment response. While we adjusted for medications using MCA, we may not have captured differences in medication use, such as adherence, dosage or combinations. Patients also had different follow-up times depending on when they were seen at outpatient visits. While we adjusted for differences in follow-up time, some medications and individuals may have required more time to garner an immunological response. Although the paired longitudinal design reduces confounding by time-invariant characteristics, it does not eliminate all potential sources of confounding, particularly for time-varying factors such as treatment. The small sample size of our study limits the ability to conduct robust sensitivity analyses, including stratifying by specific medication use, longer time between visits or other factors. Because of this, treatment effects cannot be excluded as contributors to the observed metabolomic changes. A future study with a larger sample size would improve our ability to assess these factors and better understand potential causal mechanisms. For this reason, future studies are needed to independently validate both the identity of the metabolites and robustly assess potential unmeasured confounding.

In summary, we identified numerous chemical features with abundances that changed during active SLE that were associated with remission status. Several of these were correlated with DNAm changes in immune-relevant genes and pathways. Knowledge of metabolites that change in response to treatment might serve as indicators of immune cell function and be useful for understanding long-term SLE outcomes. Further studies are needed to elucidate the potential importance of the associated metabolites, including their connection to DNAm and to develop reference panels to give meaning to identified chemical features.

## Supplementary material

10.1136/lupus-2026-002017 online supplemental file 1

10.1136/lupus-2026-002017 online supplemental file 2

## Data Availability

Data are available upon reasonable request.
