# Supplementary material for: Integrated metabolomics and DNA methylation signatures and their association with flare remission in SLE
Source: Lupus Sci Med. 2026 Sep 12;13(2):e002017. doi: 10.1136/lupus-2026-002017 (PMC13583882; doi:10.1136/lupus-2026-002017)
Supplement: Supplementary file 1 [file lupus-13-2-s001.docx]

**Supplementary Materials**

**Untargeted Metabolomics**

*Sample Extraction*

Samples were prepared by protein precipitation with cold organic solvent. 200 microliters of each sample were transferred to a clean 1.5mL microcentrifuge tubes, then 800 microliters of ice-cold methanol (Fisher Chemical, Optima™ LC-MS grade) were added. The samples were vortexed briefly (10 s) and placed in a –20°C freezer for 30 min to facilitate precipitation. Samples were subsequently centrifuged for 10 minutes at 14000 relative centrifugal force (rcf) at 4°C (Eppendorf Centrifuge 5425R). 800 microliters of supernatant, referred to as the extract, were transferred into a new 1.5 mL microcentrifuge tube and the pellet was discarded in accordance with chemical and biological safety procedures. The extract was completely dried via centrifugal evaporation (Genevac EZ-2 Plus) using the HPLC fraction setting and a maximum temperature of 40°C. The dried extracts were capped and stored at -80°C until prepared for analysis.

*Sample Resuspension*

Dried extracts were removed from storage at -80°C, allowed to warm to room temperature, and resuspended via the addition of 80 microliters of water-acetonitrile 98%:2% v/v. Resuspended extracts were briefly vortexed (15 s). An aliquot from each extract was transferred into a new 1.5 mL microcentrifuge tube to create a pooled quality control sample. An additional aliquot was transferred to a 2 mL autosampler vial with microvolume insert (Agilent).

*Analytical Measurement*

Samples were analyzed using an ultra-high performance liquid chromatograph (Vanquish, Thermo Scientific) coupled to a high-resolution mass spectrometer (Orbitrap Fusion Tribrid, Thermo Scientific). LC-MS and LC-MS/MS data were acquired. LC-MS data were collected from individual samples (n = 1 injection), system blanks (injection of solvent used to resolubilize samples), and a pooled quality control. The pooled quality control (QC) was injected multiple times at different volumes and used in data processing. LC-MS/MS data, used to annotate features, were collected using the AcquireX (Thermo Scientific) deep scan methodology in which pooled QC was injected multiple times (n = 7). Prior to measurement the mass spectrometer was calibrated using FlexMix (Thermo Scientific) following manufacture directions.

Chromatographic separation was carried out on a 2.1 x 100 mm, 100Å, 2.6 μm, F5 analytical column (Phenomenex) with corresponding guard cartridge. The column was maintained at 30°C during separation with solvent pre-heater. Gradient elution was performed after an initial period of isocratic elution using water with 0.1% acetic acid v/v (A) and acetonitrile with 0.1% acetic acid v/v (B). Separation was performed as follows: 0% B from 0 - 2.0 min, 0% to 100% B from 2.0 to 10.5 min, 100% B from 10.5 to 12.0 min, 100% to 0% B from 12.0 to 13.0 min, 0% B from 13.0 to 20.0 min. The flow rate was 0.5 mL min-1.

Ionization was performed via heated electrospray ionization (NG Ion Max, Thermo Scientific). The source parameters in positive ionization mode were as follows: spray voltage of +4000 V, sheath gas of 50 arbitrary units (arb), auxiliary gas of 10 arb, sweep gas of 1 arb, ion transfer tube at 325°C, vaporizer at 350 °C. The source parameters used in negative ionization mode were identical except for the spray voltage of -3000 V.

MS and MS/MS data were collected with an anticipated LC peak width of 8 s and a default charge of 1. EASY-IC™ (Thermo Scientific) was installed and used during data collection; a lock-mass is measured, concurrently to experimental measurement, and used for instrument mass calibration. MS data were acquired at 120,000 resolution from m/z 100-1000 with an RF lens of 60% and maximum injection time of 50 ms. MS/MS data were acquired at 30,000 resolution using an isolation width of 1.5 (m/z), stepped assisted HCD (energy steps were 20, 35, and 60, and a maximum injection time of 54 ms. The inclusion list was generated and updated via AcquireX with a low and high mass tolerance of 5 ppm. An intensity filter was applied via the “Intensity” node in the workflow with an intensity threshold of 2.0e4. The “Dynamic Exclusion” node was used with the following parameters: exclude after n = 3 times, if occurs within 15 s, exclusion duration of 6 s, a low mass tolerance of 5 ppm, a high mass tolerance of 5 ppm, and excluding isotopes.

*Untargeted Metabolomics Data Processing*

Compound Discoverer 3.3.0.550 (Thermo Scientific) was used to process.raw files which resulted in a tabular output which included descriptors of each feature (e.g. m/z, retention time), annotation information (e.g. MS/MS database match), and peak area. We processed the output from Compound Discoverer using in-house R scripts via JupyterNotebooks. The major components of the processing included formatting of the data outputs, comparison of m/z and retention time of annotation features versus an in-house generated list based on authentic chemical standards, assessment of signal response in pooled QC samples, assessment of signal variance in pooled QC samples versus samples (i.e. dispersion ratio), and multi- and univariate statistics. MSI levels of annotation confidence were provided based on the MS/MS database matching algorithm in Compound Discoverer, a list of m/z generated from authentic chemical standards, and manual annotation. (1) All features with a MS/MS database match remained at level 2 while those matching the m/z and retention time criteria were promoted to level 1.

*De Novo Annotation*

De novo annotation is described in the main body of the manuscript. Additional details are reported here for clarity and reproducibility. SIRIUS parameters for computing predicted molecular formulae:

- Instrument: Orbitrap
- Filter by isotope pattern: yes
- MS2 mass accuracy (ppm): 5
- Candidates stored: 10
- Minimum candidates per ion stored: 1
- Possible ionizations: [M+H]+ (positive mode); [M-H]- (negative mode)
- Elements allowed in molecular formula: H, C, N, O, P, S

*Weight-of-Evidence Approach for Feature Annotation*

To attain biological information about these statistically significant features where possible, we utilized the following procedure to assign a putative weight-of-evidence annotation (MSI Level 3). For all significant features with MS/MS data, data were converted to .mgf files, and probable molecular formulae were predicted in SIRIUS via fragmentation tree scoring.[[12,13]](https://www.zotero.org/google-docs/?HqvaXL) Specific parameters for computing predicted molecular formulae can be found in Supplementary Materials. Molecular formulae were also assigned based on MS1 (parent ion) matches in the Human Metabolome Database (HMDB). For each feature, the *m/z* and adduct type (M+H or M-H) were queried with a molecular weight tolerance of ±5ppm to generate a list of possible molecular formulae and corresponding possible metabolites.[[14,15]](https://www.zotero.org/google-docs/?LHWOGK) Per feature, we then compared SIRIUS-generated MS/MS molecular formula predictions and HMDB-generated MS1 molecular formula predictions. For features where SIRIUS and HMDB predictions produced the same chemical formula prediction, a consensus molecular formula was assigned to the feature (MSI Level 3). Where only a SIRIUS formula prediction or only an HMDB prediction was available, or where SIRIUS and HMDB predictions were both available but produced no consensus, no formula was assigned. For features where the putative consensus formula matched the formula and RT of an authentic reference standard, a metabolite name was assigned to the feature; these annotations were labeled with MSI Level 1 confidence.

For MSI Level 3 features assigned a consensus molecular formula (but no chemical name), the HMDB-identified metabolites for the feature formula were queried for their chemical taxonomy using ClassyFire, and a probable hierarchical chemical class was assigned to that feature based on the most-represented chemical class among possible metabolites.[[16]](https://www.zotero.org/google-docs/?KoovH3) For features where no candidate HMDB-identified metabolites had shared chemical taxonomy, no chemical class and/or subclass were assigned (represented as "no consensus"). Features thus had a final putative annotation of consensus molecular formula and, where possible, probable chemical class.

References

1. Sumner LW, Amberg A, Barrett D, Beale MH, Beger R, Daykin CA, et al. Proposed minimum reporting standards for chemical analysis Chemical Analysis Working Group (CAWG) Metabolomics Standards Initiative (MSI). *Metabolomics Off J Metabolomic Soc* 2007;3:211–221.

**Supplementary Figure.** Annotation confirmation of adenosine with authentic reference standard: m/z match and retention time match

Adenosine (*m/z* 268.1040, RT=3.34min., consensus formula C10H13N5O4) in experimental data:

Adenosine (*m/z* 268.1039, RT=3.52min.) in in-house reference library:
